# Supplementary material for: Telomere shortening causes distinct cell division regimes during replicative senescence in Saccharomyces cerevisiae
Source: Cell Biosci. 2021 Oct 9;11:180. doi: 10.1186/s13578-021-00693-3 (PMC8502270; doi:10.1186/s13578-021-00693-3)
Supplement: Supplementary file 1 — Additional file 1. Figure S1. The distribution of the timing of the first non-terminal arrest depends on the generation. Dots represent p-values of χ² goodness-of-fit tests of the null hypothesis “X following a geometric distribution (with a constant parameter)” as a function of the threshold D (see fit in Fig. 2A, red dots). Red line represents p-value = 0.05. The hypothesis is rejected for all D tested. Figure S2. A telomere-shortening model with a deterministic length threshold does not fit the experimental first non-terminal arrest data. Ordered generations of the first non-terminal arrest from the experimental data (black line, n = 115 lineages) or simulations based on the deterministic length threshold model (N = 1000), with the blue shaded area representing the 95% quantile. Figure S3. The overrepresentation of pairs of juxtaposed or closely positioned arrests is not compatible with a random distribution of arrests. Dots represent p-values of χ² goodness-of-fit tests for the distribution shown in Fig. 4A being a geometric distribution as a function of the threshold D. Red line represents p-value = 0.05. The hypothesis is rejected for most D tested. [file 13578_2021_693_MOESM1_ESM.docx]

**Additional Information**

**Title: Telomere shortening causes distinct cell division regimes during replicative senescence in *Saccharomyces cerevisiae***

**Short title:** Distinct cell division regimes leading to senescence

**Authors:** Hugo Martin^1^, Marie Doumic^1^*^,corr^, Maria Teresa Teixeira^2^* & Zhou Xu^3^*^,corr^

**Affiliations:**

^1^Sorbonne Université, JL Lions Laboratory, 75005 Paris, France

^2^Sorbonne Université, PSL, CNRS, UMR8226, Institut de Biologie Physico-Chimique, Laboratoire de Biologie Moléculaire et Cellulaire des Eucaryotes, F-75005 Paris, France

^3^Sorbonne Université, CNRS, UMR7238, Institut de Biologie Paris‐Seine, Laboratory of Computational and Quantitative Biology, 75005 Paris, France

*: Co-last authors

^corr^: Corresponding authors, [marie.doumic@inria.fr](mailto:marie.doumic@inria.fr); [zhou.xu@sorbonne-universite.fr](mailto:zhou.xu@sorbonne-universite.fr)


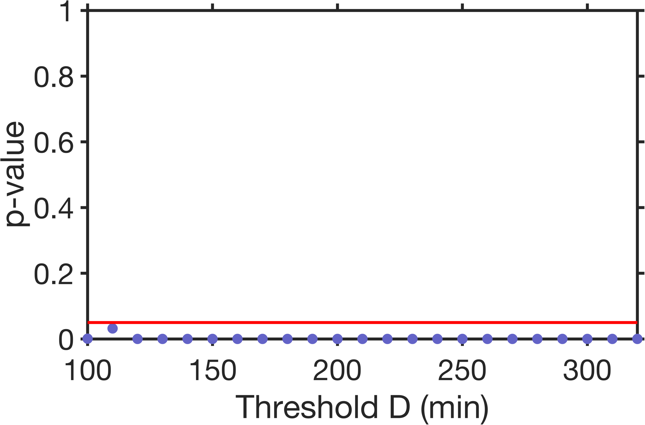


**Figure S1.** The distribution of the timing of the first non-terminal arrest depends on the generation. Dots represent p-values of χ² goodness-of-fit tests of the null hypothesis “*X* following a geometric distribution (with a constant parameter)” as a function of the threshold *D* (see fit in Fig. 2A, red dots). Red line represents p-value = 0.05. The hypothesis is rejected for all *D* tested.


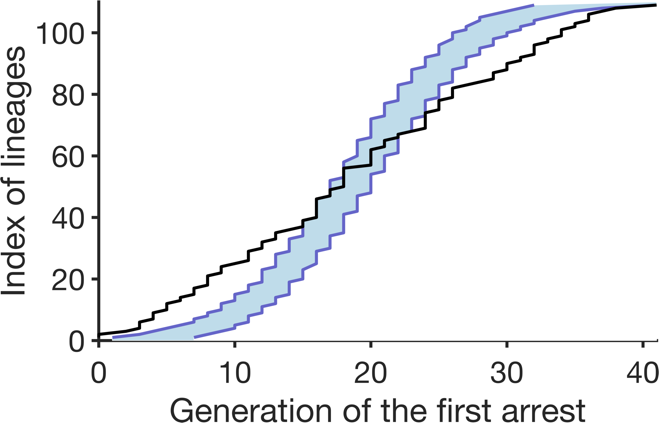


**Figure S2.** A telomere-shortening model with a deterministic length threshold does not fit the experimental first non-terminal arrest data. Ordered generations of the first non-terminal arrest from the experimental data (black line, n = 115 lineages) or simulations based on the deterministic length threshold model (N = 1000), with the blue shaded area representing the 95% quantile.


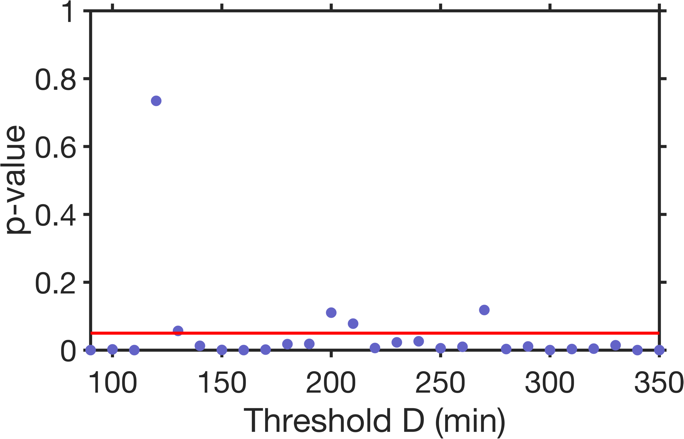


**Figure S3.** The overrepresentation of pairs of juxtaposed or closely positioned arrests is not compatible with a random distribution of arrests. Dots represent p-values of χ² goodness-of-fit tests for the distribution shown in Fig. 4A being a geometric distribution as a function of the threshold *D*. Red line represents p-value = 0.05. The hypothesis is rejected for most *D* tested.
